# Single-cell transcriptomic analysis of radiation-induced lung injury in rat

**DOI:** 10.17305/bb.2024.10357

**Published:** 2024-10-01

**Authors:** Xing-Yuan Shi, You-Qing Zhu, Chan-Jin Liang, Ting Chen, Zhi Shi, Wei Wang

**Affiliations:** 1 Department of Radiation Oncology, Nanfang Hospital of Southern Medical University, Guangzhou, Guangdong, China; 2 Department of Radiation Oncology, The Fifth Hospital of Guangzhou Medical University, Guangzhou, Guangdong, China; 3 Department of Cell Biology and Institute of Biomedicine, Guangdong Provincial Biotechnology and Engineering Technology Research Center, Guangdong Provincial Key Laboratory of Bioengineering Medicine, Genomic Medicine Engineering Research Center of Ministry of Education, MOE Key Laboratory of Tumor Molecular Biology, National Engineering Research Center of Genetic Medicine, State Key Laboratory of Bioactive Molecules and Druggability Assessment, College of Life Science and Technology, Jinan University, Guangzhou, Guangdong, China

**Keywords:** Radiation-induced lung injury (RILI), single-cell RNA sequencing (scRNA-seq), inflammatory, radiation pneumonitis (RP), cell populations

## Abstract

Radiation-induced lung injury (RILI) frequently occurs as a complication following radiotherapy for chest tumors like lung and breast cancers. However, the precise underlying mechanisms of RILI remain unclear. In this study, we generated RILI models in rats treated with a single dose of 20 Gy and examined lung tissues by single-cell RNA sequencing (scRNA-seq) two weeks post-radiation. Analysis of lung tissues revealed 18 major cell populations, indicating an increase in cell–cell communication following radiation exposure. Neutrophils, macrophages, and monocytes displayed distinct subpopulations and uncovered potential for proinflammatory effects. Additionally, endothelial cells exhibited a highly inflammatory profile and the potential for reactive oxygen species (ROS) production. Furthermore, smooth muscle cells (SMCs) showed a high propensity for extracellular matrix (ECM) deposition. Our findings broaden the current understanding of RILI and highlight potential avenues for further investigation and clinical applications.

## Introduction

Radiation therapy stands as a primary therapeutic approach in cancer treatment. However, ionizing radiation (IR) initiates DNA damage and the generation of reactive oxygen species (ROS), leading to inflammation and restructuring of the tissue. This process can result in either regeneration or restoration of organ function or persistent impairment [1–3]. The lungs are highly susceptible to IR exposure during therapeutic radiation for tumors [4, 5]. This exposure can result in inflammation that advances to clinically evident pneumonitis or fibrosis, significantly affecting patients’ quality of life [1–6]. The umbrella term for this condition is radiation-induced lung injury (RILI), encompassing both radiation pneumonitis (RP) and radiation-induced lung fibrosis (RILF) [6]. Different phases of the lung’s response to radiation exposure have been identified, categorized by the duration of exposure, comprising a total of five phases: (i) early phase, which initiates shortly after radiotherapy, spanning from hours to days; (ii) latent phase, which lasts up to four weeks after radiation exposure; (iii) the exudative phase, also known as the clinical RP phase, which typically develops between three and eight weeks after radiation exposure; (iv) intermediate phase (acute pneumonitis), which occurs 2–6 months after radiation exposure; and (v) the fibrotic phase, which may manifest after six months of radiation exposure [1, 4, 7]. These five phases are nevertheless distinct and exhibit unique characteristics. The first four phases are commonly referred to as RP. And the cytokines released during RP, especially within the initial two weeks following radiotherapy, encompass tumor necrosis factor-α (TNF-α), interleukin-1 (IL-1), interleukin-6 (IL-6), high-molecular-weight mucin-like antigen KL-6, platelet-derived growth factor-β (PDGF-β), and basic fibroblastic growth factor (bFGF) [1]. The secreted cytokines can further exacerbate the ongoing inflammatory response [8]. Studying the source of cytokines is crucial for understanding and intervening in the mechanisms and treatment of this condition. It provides insights into immune regulation, inflammation mediation, tissue repair, and potential therapeutic targets for more precise management of RILI. Furthermore, the comprehensive comprehension of the roles and molecular mechanisms of diverse cell types involved in mediating inflammatory effects remains elusive, primarily due to the reliance on bulk tissue analysis in previous studies. Analyzing the dynamically evolving heterogeneity inherent to the disease at the cellular level is constrained by the limited spectrum of flow cytometric markers employed to investigate distinct cell subpopulations in bulk samples [9]. The latest developments in single-cell RNA sequencing (scRNA-seq) offer robust capabilities for exploring the genetic and functional diversity across various cell populations, reconstructing evolutionary lineages, and identifying infrequent subpopulations [10]. This methodology has gained widespread acceptance for unraveling the molecular underpinnings of intricate conditions like COVID-19, various cancers, diabetes, Alzheimer’s disease, and more [11–16]. Therefore, utilizing single-cell sequencing technology to investigate the mechanisms of RILI and its cellular and genetic-level alterations is imperative. Given the challenges in obtaining human lung tissues affected by RILI, establishing a rat model for RILI to explore the single-cell transcriptome becomes meaningful. However, the single-cell transcriptome of rat lung tissues post-radiation remains understudied, highlighting the significance of employing single-cell sequencing technology for studying RILI in rats.

Although three studies have established mouse models of lung injury to elucidate the changes in lung tissue at the single-cell resolution during RILI, they have focused on the early phase and intermediate phase and ignored the latent and exudative phases. Ma et al. [17] established a single-cell transcriptomic profile of mouse lung tissue one-day post-radiation, revealing higher radiosensitivity in immune cells compared to stromal cells during the early phase. Yang et al. [9] studied the distinct roles played by individual cell subtypes in RILI at the 8-week post-radiation exposure during the intermediate phases. Curras-Alonso et al. constructed a murine single-cell atlas depicting the lung’s responses to radiation exposure (IR10Gy or IR17Gy) after 1–5 months. Their work unveiled a progressive series of transcriptional alterations in each lung cell population, spanning from the acute inflammatory phase to the onset of pulmonary fibrosis subsequent to exposure to a fibrogenic radiation dose [18]. The cellular and molecular changes in the latent or exudative phase can provide useful clues to the later events in RP, clinical diagnosis, and treatment of RILI. Research findings indicate that seborosteroid therapy at four weeks post-radiation improves the survival rate of mice with radiation lung injury [2]. Generally, in clinical treatment, drugs are often administered during RP. This highlights the need for a comprehensive exploration of the latent and exudative period to achieve a more thorough understanding of RILI dynamics and treat RILI. Moreover, previous researches have primarily concentrated on elucidating alterations in epithelial and endothelial cells, given their prominent and feasible changes observed in tissue experiments [1, 4]. However, other cell types, including neutrophils, macrophages, monocytes, and smooth muscle cells (SMCs) also contribute significantly to the progression of RILI. In addition, in some clinical cases, higher doses of radiation may be required, and RILI relies on the radiation dose, and higher doses of radiation may accelerate the occurrence and progression of radiation lung injury. However, the majority of prior research utilized the animal radiation model within the range of 10–15 Gy, with less emphasis on the impact of higher doses. Therefore, we chose a time point (two weeks post-radiation) and subjected rats to a thoracic radiation dose of 20 Gy. This approach was adopted to mimic the radiation damage caused by a single exposure to 20 Gy, in the establishment of the rat model for RILI. Simultaneously, leveraging single-cell sequencing technology, we acquired the single-cell transcriptome data of lung tissues from rats irradiated after a two-week post-radiation period and those not exposed to radiation. This analysis aimed to elucidate cellular and molecular changes in lung tissues, particularly focusing on cell populations characterized by heightened inflammatory responses, elevated cytokine levels, and enhanced intercellular communication. The insights gained from this study aim to contribute valuable knowledge to the understanding of the intricate cellular responses involved in RILI and provide valuable references for precise treatments of RILI, ultimately enhancing the effectiveness and precision of RILI treatment protocols, especially in terms of cell-specific therapies for RP and the screening of targeted drugs.

## Materials and methods

### Rats

Four female SD rats (weight: 160 ± 10 g) and four male SD rats (weight: 190 ± 10 g) at six weeks old with specific weights were purchased from Guangzhou Ruige Biotechnology Co., Ltd., (Guangzhou, China) and were kept within Guangzhou Medical University animal facilities. All rats were raised in a clean environment with a consistent dark-light schedule (lights on from 7 a.m. to 7 p.m.). This study included four rats (two female SD rats and two male SD rats) that received thoracic radiation, while the other four (two female SD rats and two male SD rats) were randomly selected as negative controls. Four irradiated rats were sacrificed at two weeks post-radiation (here called IR20Gy group) and consequently, lung tissues were harvested intact.

### Radiation injury

Four rats were sedated with an intraperitoneal dose of 0.3% Sodium pentobarbital (40 mg/kg body weight), fixed in a position supine, and placed vertically at 500 mm from an electron beam. The rats were then subjected to bilateral thorax radiation (from the clavicle to the lower margin of the costal arch) with a single dose of 20 Gy at eight weeks old utilizing the 4.5-MeV linear electron accelerator facility (VARIAN Trilogy) to induce lung injury. These four rats were referred to as the IR20Gy group.

### Computed tomography (CT) scan

The lung was imaged with computed tomography (CT) (Aquilion ONE TSX-301C, Canon, Japan) before being sacrificed. Before imaging, the rats were anesthetized with 0.3% sodium pentobarbital (40 mg/kg body weight) and placed in a supine position. All imaging analyses were performed by two radiologists independently.

### Single-cell suspension preparation

Two irradiated rats and two control rats were selected for preparation, and lung tissues were extracted for conducting scRNA-seq. Remove lung tissue and place it in a 6-well plate containing pre-chilled PBS for washing, striving to remove necrotic tissue, blood, and other impurities. Transfer the tissue to a 1.5-mL centrifuge tube designed for low binding, add 1 mL of digestion solution, and then proceed to mince the tissue using scissors. Place the tube on a rotating shaker in a 37 ^∘^C incubator, rotating at approximately 20 rpm for 10 min. Remove the centrifuge tube and pipette up and down 30 times, then return it to the rotating shaker in the 37 ^∘^C incubator for an additional 10 min of digestion. After digestion, remove the centrifuge tube and pipette up and down 30 times, and then conduct acridine orange/propidium iodide (AOPI) quality control (QC) (1:1). Use a 70 µm cell strainer to sieve the tissue, and the centrifuge at 400G for 5 min at 4 ^∘^C. Discard the filtrate and resuspend the sediment in 1 mL of 1640 medium, and then conduct AOPI QC (1:1). Add 3 mL of trypan blue solution and incubate for 2 min. Terminate trypan blue staining by adding 4 mL of 1640 medium. Centrifuge at 400 G for 5 min at 4 ^∘^C. Mix the precipitate in 1640 medium, adjusting the concentration to 600–1500 cells/µL.

### Single-cell RNA library construction and sequencing

The cell suspension was introduced into microfluidic chips equipped with 3’ chemistry, followed by barcoding using a 10× Chromium Controller (10× Genomics). Subsequently, RNA from the barcoded cells underwent reverse transcription, and sequencing libraries were prepared utilizing reagents from a Chromium Single Cell 3’ v2 reagent kit (10× Genomics), adhering to the manufacturer’s guidelines. Sequencing was performed at the Chabiotech Co., Ltd., (Shenzhen, China) on an Illumina platform (HiSeq 2000) as per the protocols provided by Illumina.

### Hematoxylin–eosin (H&E) stain

The tissue specimens were obtained from eight SD rats, including two irradiated rats two weeks post-radiation, two unirradiated rats, and the residual lung tissue taken during single-cell suspension preparation. The selected tissue section was encased in paraffin and subjected to hematoxylin–eosin (H&E) staining as outlined. The sections were then rehydrated with xylene and alcohol and stained with hematoxylin (MACKLIN, C11915133, China) and eosin (SCR, 20210812, China) to label the nucleus and cytoplasm. Subsequently, the tissue specimens were examined under a microscope (BX53, Olympus, Japan). Two independent pathologists examined at least five views per rat for all histological analyses.

### Preprocessing scRNA-seq data

We utilized the Cell Ranger software by 10× Genomics to demultiplex the raw data files, extracting barcodes, and aligning the reads obtained from droplet-based scRNA-seq to the reference rat genome. This process resulted in the generation of gene expression matrices representing unique molecular identifier (UMI) counts per gene per cell for each sample. Subsequently, we employed standard Seurat (version 5.0.1) R toolkits to integrate the expression matrices obtained from the four samples for downstream analyses. We applied Harmony [19] to mitigate batch effects and conducted data normalization, scaling, downstream dimensionality reduction, and clustering. During QC procedures, genes expressed in fewer than three cells and cells with expression levels below 200 or above 5000 genes (partly sample above 4500 genes), as well as those with over 15% UMIs derived from the mitochondrial genome, were excluded. As a result, 24,323 cells were retained for subsequent analysis. Using the FindClusters function with default parameters and a resolution parameter set to 0.2, we identified 18 clusters representing different cell subtypes.

### Cell-type annotation

The ScType [20] tool was initially employed for unbiased cell annotation, followed by manual adjustments for precision. These adjustments involved integrating established markers from the literature for each cell cluster and incorporating functional analysis results from Gene Ontology (GO) functional enrichment analysis of differentially expressed genes (DEGs) within each cluster. Additionally, differential genes identified by the FindAllMarkers function were utilized for GO functional enrichment analysis. The final cell annotation was achieved through manual correction, which combined established markers from published literature and GO analysis results derived from FindAllMarkers (the parameter was set by only.pos ═ TRUE, min.pct ═ 0.2, logfc.threshold ═ 0.25).

### Gene set score

The corresponding mouse gene sets termed ‘HALLMARK_INFLAMMATORY_RESPONSE’, ‘HALLMARK_EPITHELIAL_MESENCHYMAL_TRANSITION’, ‘HALLMARK_APOPTOSIS’ and ‘HALLMARK_REACTIVE_OXYGEN_SPECIES_PATHWAY’ respectively, from MSigDB were downloaded [21]. Extracellular matrix (ECM) genes, transdifferentiation genes, and cytokine genes were collected based on references [18, 22]. Cytokine, ECM, transdifferentiation, inflammatory, epithelial–mesenchymal transition (EMT), apoptosis, and ROS score were assessed using the AddModuleScore function integrated within Seurat [23]. Since these gene sets are derived from mice or humans, we utilized the “homologene” package to ascertain orthologous genes in rats. Subsequently, these identified genes were compiled and included in the Table S1. We performed a *t*-test on the score in the control and IR20Gy group and the results were shown in Table S2.

### DEG acquisition

FindMarkers was employed to detect DEGs between the control and IR20Gy groups. We studied the genes exhibiting an adjusted *P* value below 0.05, indicating whether a group of genes demonstrates statistically significant, consistent variations between two conditions. Additionally, a set of genes with log2FC >0 were defined as upregulated genes and conversely, log2FC <0 were downregulated genes.

### Enrichment analyses

GO and Kyoto Encyclopedia of Genes and Genomes (KEGG) enrichment analyses were performed using the “clusterProfiler” R package [24, 25]. This analysis aimed to identify biological processes or pathways significantly associated with the DEGs. Biological processes or pathways with *P* < 0.05 were deemed significantly enriched.

### Analysis of cell–cell interaction

Exploration of potential cell–cell communications following radiation exposure was conducted using CellChat [26]. We utilize pathways related to three modes of cell interaction, namely, secreted signaling, cell–cell contact, and ECM-receptor as the CellChat receptor ligand database. This database serves as the foundation for our analysis of cell interactions. Following the official tutorial, we initially conducted cell interaction analysis on a single dataset and subsequently extended our analysis to multiple datasets.

### Ethical statement

The animal experiments were specifically approved by the Ethics Committee of Guangzhou Medical University (G2023-781).

### Statistical analysis

We performed a *t*-test on the inflammatory, cytokine, ECM, EMT, apoptosis, ROS, and transdifferentiation score in the control and IR20Gy group and the results were shown in supplementary material Table S2. In the R environment, the analysis of transcriptomic data at the single-cell level was conducted using a range of R tools, with statistical significance set at *P* < 0.05, employing the default corresponding statistical methods provided by the packages.

## Results

### Changes in cell composition and functions

**Figure 1.**
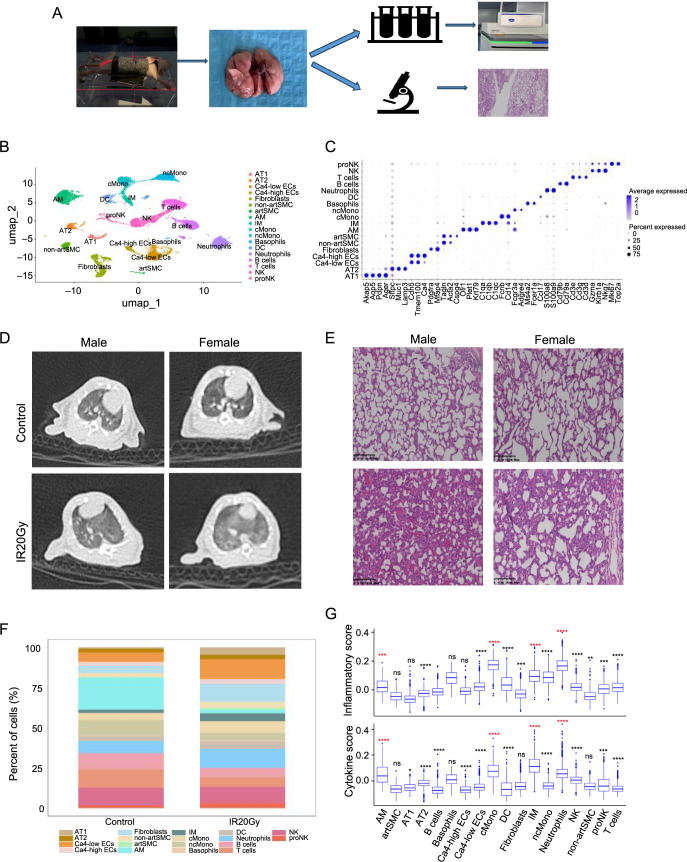
**Composition and function changes in RILI cells.** (A) Scheme of the experimental setup. Rats were irradiated with a dose of 20 Gy and sacrificed at two weeks after IR, and lungs were enzymatically and mechanically dissociated into a single cell suspension before being loaded in the 10× Chromium Controller System; (B) UMAP visualization of 24,323 cells from four different samples (two samples from control group; two samples from IR20Gy group) annotated by cell type; (C) Dot plot of the expression of the markers used for cell-type identification; (D) Displayed are axial images from CT scans of lung tissues; (E) Displayed are H&E staining results of representative sections; Scale bar represents 200 µm; (F) Dynamics in cell proportions of the 18 cell types; (G) The inflammatory and cytokine scores of each cell type in the IR20Gy group. The cell type of which *P* value was marked with red got increasing and high inflammatory and cytokine scores. ns, *P* > 0.05; *P <0.05; **P <0.01; ***P <0.001; ****P <0.0001. AT1: Alveolar epithelial type I; AT2: Alveolar epithelial type II; artSMC: Arterial smooth muscle cells; non-artSMC: Non-arterial smooth muscle cells; Ca4-high ECs: Ca4 high endothelial cells; Ca4-low ECs: Ca4 low endothelial cells; cMono: Classical monocytes; ncMono: Non-classical monocytes; AM: Alveolar macrophages; IM: Interstitial macrophages; DC: Dendritic cells; NK: Natural killer cells; proNK: Proliferating natural killer cells; UMAP: Uniform manifold approximation and projection; RILI: Radiation-induced lung injury; H&E: Hematoxylin–eosin.

To assess the pulmonary effects of radiation exposure, we conducted scRNA-seq utilizing the 10× Chromium Controller v2 platform. We analyzed dissociated lung tissues from the control group of unirradiated rats and another group of rats subjected to a single 20-Gy dose of IR two weeks prior (referred to as the IR20Gy group) (Figure 1A). A total of 26,700 cells were obtained, with 13,316 belonging to the control group and 13,384 to the IR20Gy group. After QC, a total of 24,323 cells were included for subsequent analysis. The analysis of scRNA-seq data identified 18 cell types, characterized by marker genes from recent literature [9, 17, 18, 27–31]. These included two epithelial cell clusters (alveolar epithelial type I [AT1] and type II [AT2]), three mesenchymal cell clusters (fibroblasts, arterial SMCs [artSMC], and non-arterial SMCs [non-artSMC]), two endothelial cell clusters (Ca4 high endothelial cells [Ca4-high ECs] and Ca4 low endothelial cells [Ca4-low ECs]), seven myeloid cell clusters (classical monocytes (cMono), non-classical monocytes (ncMono), alveolar macrophages (AM), interstitial macrophages (IM), dendritic cells (DC), neutrophils, and basophils), and four lymphoid cell clusters (T cells, B cells, natural killer cells [NK], and proliferating natural killer cells [proNK]) (Figure 1B and 1C). To distinguish individual clusters and ascertain their respective markers, we performed a differential analysis using the FindAllMarkers function across all cell clusters. A heatmap was generated using the top ten DEGs for each cellular subtype (Figure S1A). These genes exhibited distinctly elevated expression levels in each cluster, with some genes not previously identified as specific markers for certain cell types. This discovery introduces novel markers for the precise identification of specific cell populations, such as Slc39a2, which could be used to mark AM, and Postn, which could be used to mark artSMC.

To investigate alterations in the cell composition of irradiated rat lung tissues, our aim was to detect alterations in the proportions of distinct cell populations post-radiation. The results, depicted in Figure 1F and Figure S1B, revealed a declining trend in the proportions of immune cells and an increasing trend in the proportions of other cells after radiation. Notably, AM experienced the most significant reduction, while among non-immune cells, AT1, Ca4-low ECs, and fibroblasts exhibited a more pronounced increasing trend. However, IM, cMono, neutrophils, DC, and proNK showed an increased proportion.

To assess the pathological alterations in rat lungs, CT scans were performed on both control and IR20Gy groups. The results of CT scans revealed ground-glass opacities with a hazy appearance and reduced density, indicative of pneumonia-like changes in the IR20Gy group (Figure 1D). Additionally, the histopathological analysis using H&E staining exhibited prominent inflammatory responses in the lung tissue sections of the IR20Gy group (Figure 1E). Subsequently, to explore the role of individual cell subpopulations in radiation pneumonia and potential cytokine production sources, we calculated inflammatory and cytokine scores for each cell using the expression level of collected inflammatory response and cytokine genes, respectively (Figure 1G). The results demonstrated a noticeable upregulation of inflammatory and cytokine gene expression in the IR20Gy group, indicating the presence of radiation pneumonia. Specifically, four cell subtypes, including AM, IM, neutrophils, and cMono, exhibited increased and high inflammatory and cytokine scores after radiation. These findings suggest that these four cell subtypes may play a pivotal role in radiation pneumonia and serve as primary sources of cytokine release.

To identify genes exhibiting significant expression differences and potentially associated with the onset, progression, or other relevant biological processes, we conducted differential gene expression analysis comparing the control and IR20Gy groups with FindMarkers. Genes with an adjusted *P* value <0.05 and a log2FC > 0 were considered upregulated, while those with an adjusted *P* value < 0.05 and a log2FC < 0 were considered downregulated in the IR20Gy group compared to the control group. The results were visualized in a volcano plot, with the top ten differentially upregulated and downregulated genes in the IR20Gy group marked in black (Figure S1C). The changes in their expression levels post-radiation are depicted using violin plots and dot plots in Figure S1J–S1M. Among these, ENSRNOG00000064228 and RGD1563400, officially known as *Pilrb-ps6* and *Pilrb2l2*, respectively, emerged as the top two upregulated genes in the IR20Gy group. These genes encode transmembrane receptor proteins expressed on myeloid lineage cells. *Pilrb* belongs to the Siglec family of receptors and exhibits primarily expression on cells of the myeloid lineage. It has been implicated in modulating inflammatory responses within the innate immune system [32–34].

To elucidate the associated biological processes and pathways linked to the upregulated and downregulated genes, we performed enrichment analyses using GO and KEGG (Figure S1D, S1E, S1H, and S1I). The upregulated genes exhibited enrichment in diverse KEGG pathways, indicating their involvement in processes, such as neurodegeneration, infection response (human papillomavirus), cell signaling (PI3K/Akt, Hippo), cellular structure (focal adhesion), protein processing, atherosclerosis, and small cell lung cancer (Figure S1D). The downregulated gene enrichment results encompassed various KEGG pathways related to immune response, viral infection, and cellular metabolism (Figure S1E). This suggests possible immune system suppression as a distinctive trait within the studied context. Additionally, the results of GO enrichment analysis, focusing on biological process (BP) enrichment analysis, revealed that upregulated genes were associated with processes like wound healing, renal system development, cell–substrate adhesion, ECM organization, and extracellular structure organization (Figure S1H). On the other hand, downregulated genes were mainly associated with immune response, encompassing the activation and regulation of signaling pathways pertinent to immune response (Figure S1I). These findings suggest that radiation exposure may weaken the immune function of rat lung tissues, thereby influencing the initiation and advancement of inflammation.

To identify cytokines involved in the inflammatory response among the upregulated and downregulated genes, we utilized a Venn diagram to extract overlapping genes. The results were presented in Figure S1F and S1G. There were 98 common genes between upregulated genes and inflammatory response genes (Figure S1F). And the overlapping genes among upregulated genes, inflammatory response genes, and cytokine genes included 14 genes *Ccl7*, *Csf1*, *Cxcl10*, *Cxcl11*, *Cxcl9*, *Ebi3*, *Hbegf*, *Il10*, *Il15*, *Il1a*, *Il1b*, *Il6*, *Lif,* and *Tnfsf9*. The alterations in their expression levels post-radiation across each cell population are illustrated using violin plots and dot plots in Figure S1N–S1Q. These genes may play a pivotal role in RILI, potentially acting as either anti-inflammatory or proinflammatory factors. Moreover, there were 23 shared genes between downregulated genes and inflammatory response genes, along with two overlapping genes *Il18* and *Ccl5* (Figure S1G). Violin plots and dot plots in Figure S1R–S1U depict the changes in their expression levels across each cell population post-radiation.

### Increased cellular interaction quantity and intensity

**Figure 2.**
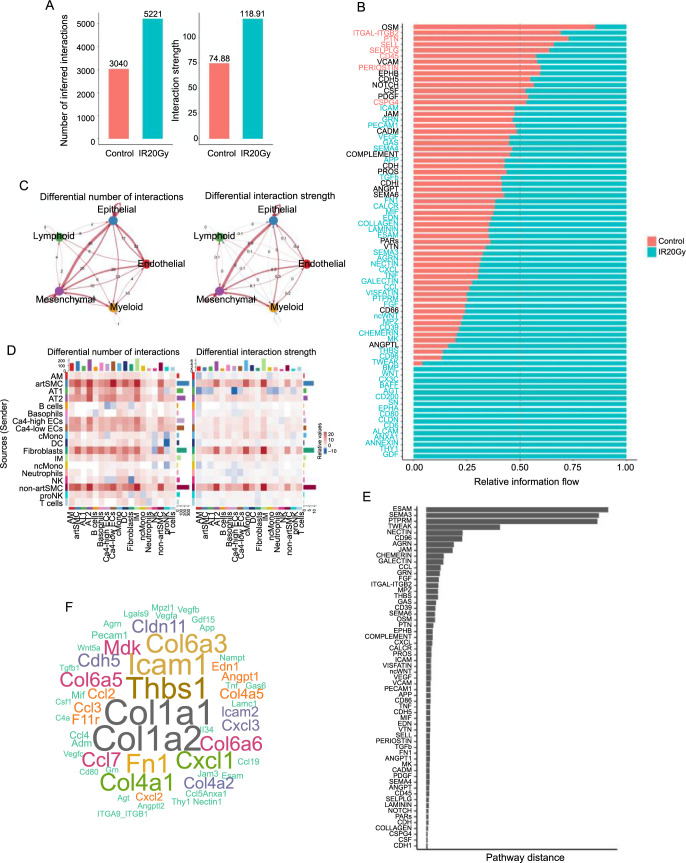
**Cell–cell interaction analysis between the different lung cell populations after radiation.** (A) Circle plot showing the differential number of interactions and strength: mesenchymal, endothelial, epithelia, myeloid, and lymphoid. Red (or blue) colored edges represent increased (or decreased) signaling in the IR20Gy group compared to the control group. (B) All significant signaling pathways were ranked based on their differences in overall information flow within the inferred networks. The colored red signaling pathways are more enriched in the control group, the colored black ones are equally enriched in both control and IR20Gy groups, and the colored green ones are more enriched in the IR20Gy group. (C) The circle graph shows the changes in the number and intensity of the five cell populations (myeloid, endothelial, epithelial, lymphoid, mesenchymal) (indicated by an increase in quantity or intensity in red, decrease in blue). (D) Comparison of the number and strength changes in 18 clusters by heatmap (indicated by an increase in quantity or intensity in red, decrease in blue). The top bar graph of the heatmap represents the number or strength of signals received by the corresponding cell type, and the right bar graph represents the number or strength of signals emitted by the corresponding cell type. (E) The overlapping signaling pathways were ranked based on their pairwise Euclidean distance in the shared two-dimensional manifold according to their functions. A larger distance implies a larger difference in functions. (F) The enriched ligands in the IR20Gy group shown by using word cloud. IR20Gy: Ionizing radiation exposure at a dose of 20 Gray.

To anticipate the cellular changes crucial for RILI development, we examined the dynamic evolution of potential cell–cell communications post-radiation. Employing CellChat, we identified cell types likely interacting based on scRNA-seq data, ligand–receptor expressions, and their specific moments of interaction (Figure 2A). Our analysis revealed an amplified number and strength of cell–cell communications after radiation. Specifically, the interactions between epithelial cells and mesenchymal cells, mesenchymal cells and myeloid cells, mesenchymal cells and endothelial cells, along with epithelial cells and endothelial cells exhibited heightened numbers and strength after radiation compared to the control group (Figure 2C). Subsequent detailed analysis indicated an overall increased communication among almost all cell types in the IR20Gy group, with a notable emphasis on interactions among artSMC, non-artSMC, fibroblasts, IM, cMono, and AT2 (Figure 2D and Figure S2A and S2B).

Next, we compared the flow of information for each signaling pathway between the control and IR20Gy group. The flow of information within a specific signaling pathway is determined by the cumulative communication probability among all pairs of cell groups in the inferred network [26]. We observed that certain pathways maintained similar flow between the two groups, such as OSM, VCAM, NOTCH, CSF, and SEMA6 (black in Figure 2B). On the contrary, there are notable changes in the information flow within specific pathways in the IR20Gy group as compared to the control group: (i) decrease (in ITGAL-ITGB2, PTN, and SELL), (ii) turn on (in BMP, WNT, GDF, and THY1), or an (iii) increase (in VEGF, SEMA4, TGFb, MIF, COLLAGEN, SEMA3, tumor necrosis factor [TNF], and TWEAK). Subsequently, through the computation of the Euclidean distance between each pair of common signaling pathways within the shared two-dimensional manifold, we noted substantial distance for signaling pathways such as ESAM, SEMA3, PTPRM, and TWEAK (Figure 2E), indicating significant variations in the network architectures of these pathways. In contrast, several other signaling pathways exhibit comparatively minor distances, such as COLLAGEN, CSPG4, CSF, and CDH1 (Figure 2E). This implies comparable communication network structures for these shared pathways in both groups. Furthermore, for elucidating the coordinated functioning of multiple cell groups and signaling pathways, CellChat was employed to characterize the global communication patterns and identify key signaling events within distinct cell groups (Figure S2C and S2D). The results uncovered three patterns for incoming signaling (Figure S2C) and two patterns for outgoing signaling (Figure S2D). For instance, the analysis indicated that a significant portion of incoming neutrophil signaling corresponded to pattern 1, which represented various pathways, encompassing but not limited to MIF, MK, and TNF (Figure S2C). The outgoing signaling from the immune cell was characterized by pattern 1, representing such pathways as CXCL, CSF, SELL, and TNF. Furthermore, to identify the upregulated signaling in IR20Gy, we performed the comparison of the significantly upregulated ligand–receptor pairs between the control and IR20Gy groups based on the upregulated genes. Figure S2E and S2F demonstrates that App-Cd74 was identified as a significant ligand–receptor pair after radiation, with Cxcl2-Cxcr2 emerging as the most crucial signaling molecule in Myeloid-to-Myeloid communication (Figure S2E and S2F). From a word cloud analysis (Figure 2F), we identified Col1a1, Col1a2, and Thbs1 as the top enriched ligands in the IR20Gy group.

### Subtypes of neutrophils show inflammatory effects following radiation

**Figure 3.**
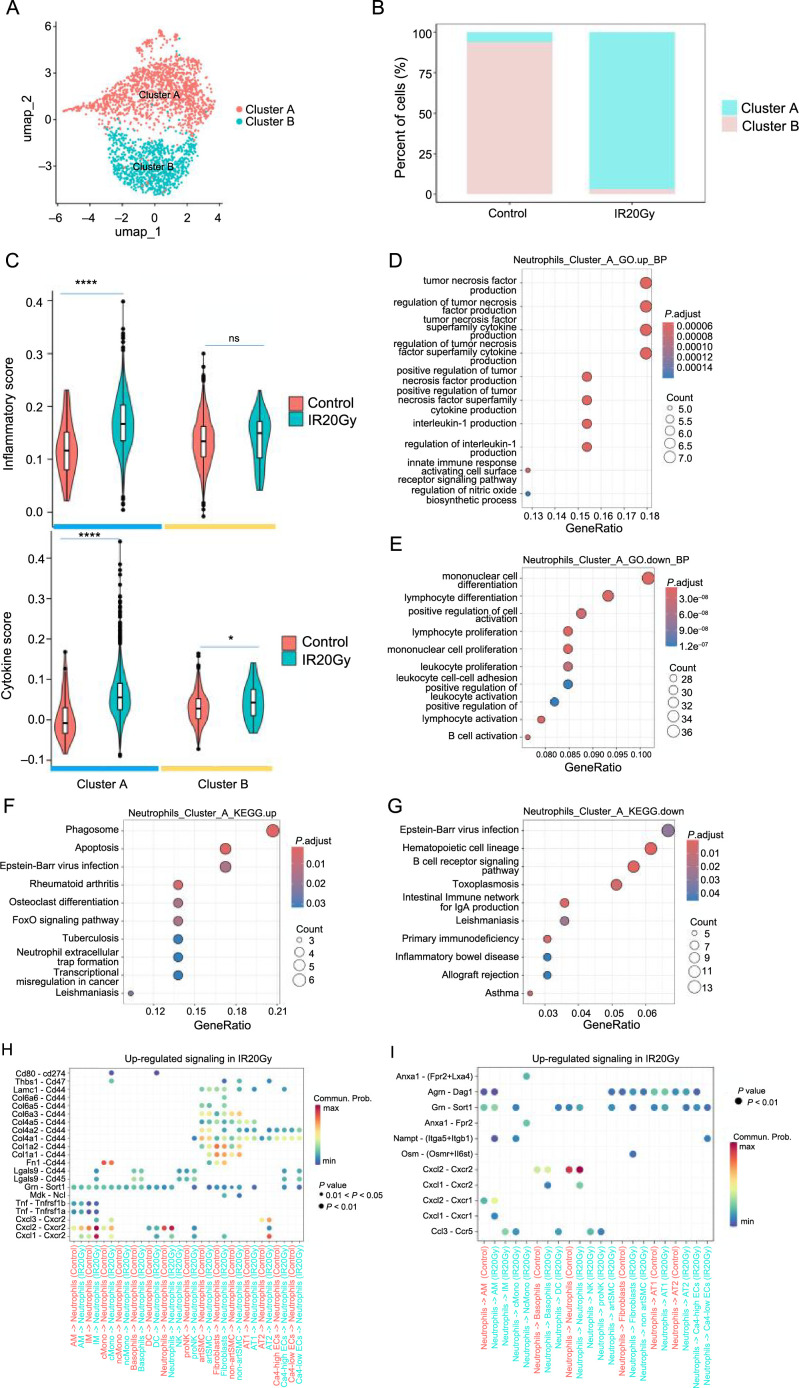
**Cellular and molecular changes in the neutrophil cells reveal proinflammatory neutrophil subpopulations after radiation.** (A) UMAP visualization of the different neutrophil cell subpopulations; (B) Changes in cell proportions of neutrophil subpopulations; (C) The inflammatory and cytokine score of the two subpopulations of neutrophils; (D and E) GO terms enriched with the cluster A differential genes in the IR20Gy group compared to the control group; (D) Upregulated; (E) Downregulated; (F and G) KEGG enriched with the cluster A DEGs in the IR20Gy group compared to the control group; (F) Upregulated; (G) Downregulated; (H) Upregulated signaling associated with upregulated genes in the IR20Gy group received by neutrophils; (I) Upregulated signaling associated with upregulated genes in the IR20Gy group sent by neutrophils. ns, *P >*0.05; *P <0.05; ****P <0.0001. UMAP: Uniform manifold approximation and projection; KEGG: Kyoto Encyclopedia of Genes and Genomes; IR20Gy: Ionizing radiation exposure at a dose of 20 Gray; GO: Gene Ontology; DEGs: Differentially expressed genes.

Neutrophils play a pivotal role in lung physiology, participating in the clearance of infections through phagocytosis, mediating inflammation, and contributing to tissue repair [27, 35, 36]. Their rapid response and immune-modulating functions contribute to lung defense and homeostasis. A detailed analysis of neutrophils compartment across all lung samples distinguished two clusters (Figure 3A): cluster A expressed Cc13, Mgp, and Ccl2, and cluster B expressed Fos, Sik1, Oas12, and Rgs2 (Figure S3H). Notably, the distribution of neutrophils within these subpopulations was not uniform across all samples. Cluster A was dramatically enriched after radiation, while cluster B was the opposite (Figure 3B).

We generated an inflammatory and cytokine score based on a gene set downloaded from MSigDB and collected cytokine genes. A significant increase in the score was in cluster A in the IR20Gy group, while nonsignificant change was in cluster B, indicating that cluster A may play a key role in inflammation (Figure 3C). Interestingly, the overlapping genes of the neutrophil-upregulated genes and inflammatory genes are enriched in cluster A, while those of downregulated and inflammatory genes are enriched in cluster B (Figure S3I–S3L). The results of GO and KEGG analyses indicated the enrichment of upregulated genes in neutrophils in processes, such as apoptosis, oxidative phosphorylation, and NF-kappa B signaling pathway, while the downregulated genes in neutrophils were associated with osteoclast differentiation and mechanical stimulus–response (Figure S3A–S3F). The KEGG analysis of cluster A revealed that radiation may cause changes in immune response, cell apoptosis, and inflammation (Figure 3F and 3G). The GO enrichment analysis results for upregulated genes reveal significant enrichment in well-known proinflammatory factors production, such as TNF, IL-1, and the TNF superfamily cytokine (Figure 3D). Notably, it is worth highlighting that there is also enrichment observed in the positive regulation of TNF production and TNF superfamily cytokine production (Figure 3D), suggesting a heightened regulatory activity specifically targeting the generation of TNF and TNF superfamily cytokine. Enriched GO terms for downregulated genes primarily highlight immune cell proliferation, encompassing leukocytes, mononuclear cells, and lymphocytes (Figure 3E). Additionally, there is a significant enrichment observed specifically in GO terms associated with the positive regulation of immune cell activation, suggesting a pronounced downregulation of immune function (Figure 3E). In summary, our inference indicates that following radiation exposure, Cluster A promotes the release of TNF and TNF superfamily factors while inhibiting the activation and proliferation of immune cells, resulting in diminished immune function and heightened inflammatory response. Moreover, interestingly, the two genes, *ENSRNOG00000064228* and *RGD1563400*, which have been implicated in modulating inflammatory responses within the innate immune system, were also the top two upregulated genes in neutrophils after radiation (Figure S3G). Additionally, among all upregulated signaling ligand–receptor pairs in neutrophils, Cxcl1-Cxcr2 and Cxcl2-Cxcr2 showed the highest communication probability sent by IM (Figure 3H and 3I).

### Specific macrophage compartments display stronger inflammatory response and cytokine release profiles after radiation

**Figure 4.**
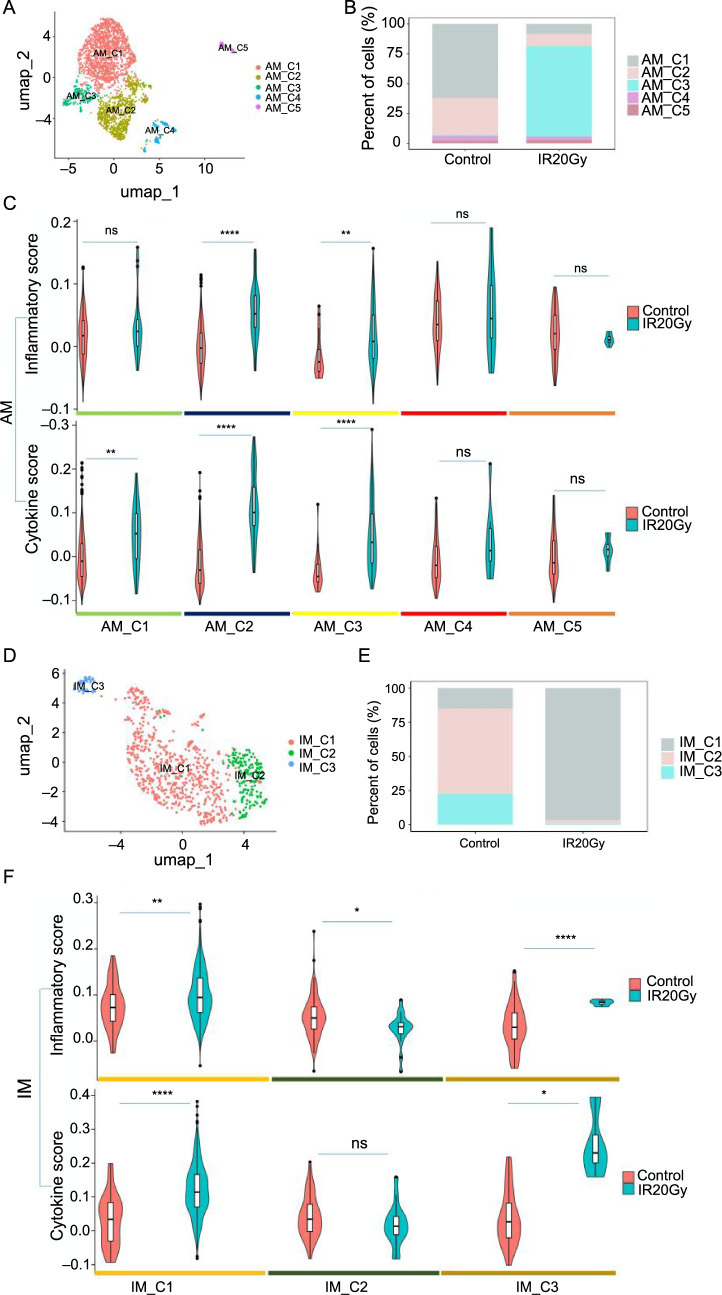
**Increased inflammatory response and cytokine release profile of alveolar and IMs after radiation.** (A–C) Cellular and molecular characterization of AM; (A) UMAP visualization of the different AM subpopulations; (B) Changes in cell proportions of AM subpopulations; (C) The inflammatory and cytokine score of the five subpopulations of AM; (D–F) Cellular and molecular characterization of IM; (D) UMAP visualization of the different IM subpopulations; (E) Changes in cell proportions of IM subpopulations; (F) The inflammatory and cytokine scores of the three clusters of IM. ns, *P >* 0.05; *P <0.05; **P <0.01; ****P <0.0001. UMAP: Uniform manifold approximation and projection; IM: Interstitial macrophages; AM: Alveolar macrophage.

Macrophages serve crucial functions in repairing lung injuries, safeguarding the host against pathogens, managing inflammatory conditions, maintaining tissue equilibrium, and facilitating remodeling processes [35, 37, 38]. We identified two macrophage subpopulations (Figure 1B and 1C): AM-expressed Olr1, Krt79, and Plet1 (Figure 1C), and IM-expressed C1qa, C1qb, and C1qc (Figure 1C). Additional investigation revealed that AM could be categorized into five distinct subpopulations (Figure 4A and Figure S4A). Similarly, IM was distributed into three different subpopulations (Figure 4D and Figure S4F). The distribution of macrophages across these subpopulations was not uniform across all samples. For example, AM_C3 and IM_C1 subpopulations were enriched in the IR20Gy group (Figure 4B and 4E). Furthermore, we conducted inflammatory and cytokine scoring on macrophages. Following IR, AM_C2, AM_C3, IM_C1, and IM_C3 consistently exhibited significantly elevated scores for both inflammation and cytokine levels. (Figure 4C and 4F). These cellular subtypes exhibit heightened inflammatory responses and cytokine release, suggesting a potential propensity for exaggerated immune reactions that may contribute to inflammation.

**Figure 5.**
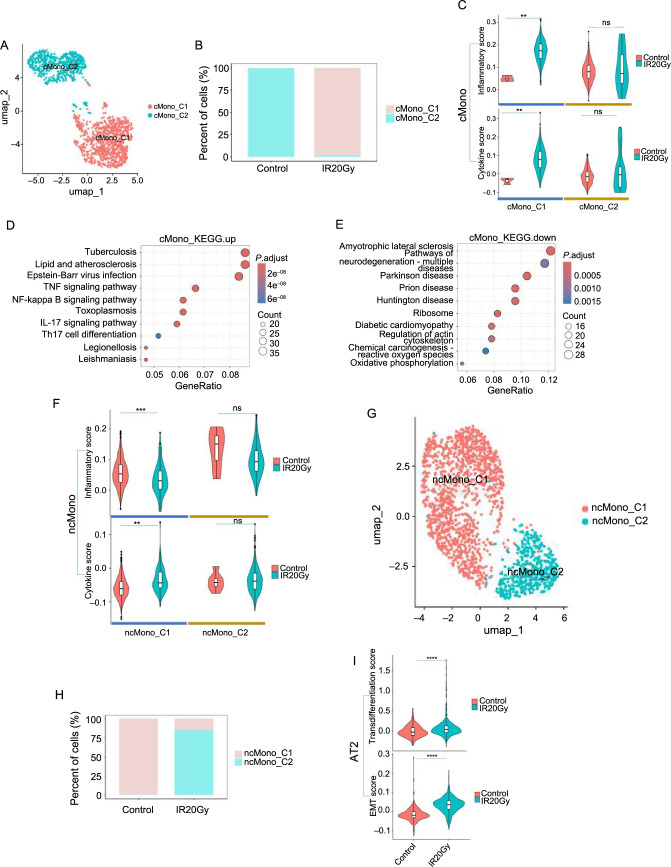
**Cellular and molecular characterization of monocytes and AT2.** (A–E) Specific cMono subtype shows a proinflammatory profile after IR20Gy; (A) UMAP visualization of the different cMono subpopulations; (B) Changes in cell proportions of cMono subpopulations; (C) The inflammatory and cytokine score of the two subpopulations of cMono; (D and E) KEGG pathways enriched by the DEGs of cMono in the IR20Gy group compared to the control group; (D) Upregulated; (E) Downregulated; (F–H) Cellular and molecular characterization of ncMono; (F) The inflammatory and cytokine score of the two clusters of ncMono; (G) UMAP visualization of the different ncMono subpopulations; (H) Changes in cell proportions of ncMono subpopulations; (I) The transdifferentiation and EMT score in AT2. ns, *P >*0.05; **P <0.01; ***P <0.001; ****P <0.0001. UMAP: Uniform manifold approximation and projection; IR20Gy: Ionizing radiation exposure at a dose of 20 Gray; AT2: Alveolar epithelial type II; DEGs: Differentially expressed genes; KEGG: Kyoto Encyclopedia of Genes and Genomes; cMono: Classical monocytes; ncMono: Non-classical monocytes.

**Figure 6.**
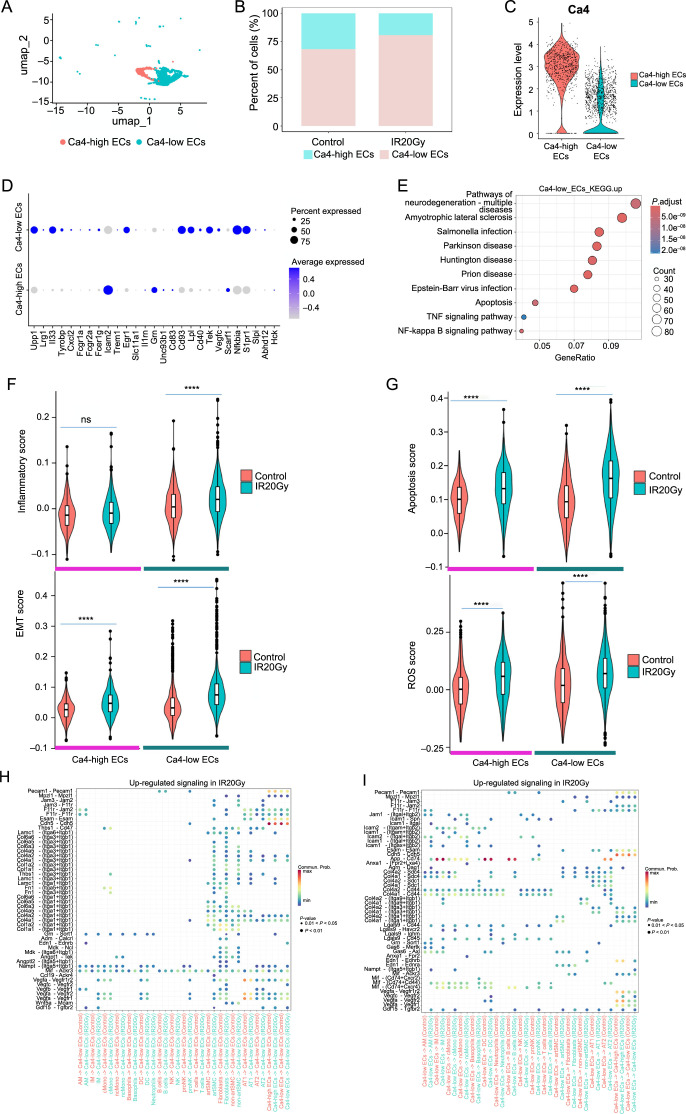
**Cellular and molecular characterization of endothelial cells.** (A) UMAP visualization of the two subpopulations of endothelial cells (Ca4-high ECs and Ca4-low ECs); (B) Changes in cell proportions of Ca4-high ECs and Ca4-low ECs in ECs; (C) The Ca4 expression in the two cell types; (D) Dot plot shows the expression of the genes related to neutrophil recruitment and inflammatory response in the two EC subsets; (E) KEGG enrichment analysis of the Ca4-low ECs upregulated genes in the IR20Gy group compared to the control group. (No significant KEGG pathway was enriched by the downregulated genes in Ca4-low ECs); (F) The inflammatory and EMT score of the two cell types in each group; (G) The apoptosis and ROS scores of the two cell types; (H) Upregulated signaling associated with upregulated genes in the IR20Gy group received by Ca4-low ECs; (I) Upregulated signaling associated with upregulated genes in the IR20Gy group sent by Ca4-low ECs. ns, *P >*0.05; ****P <0.0001. UMAP: Uniform manifold approximation and projection; IR20Gy: Ionizing radiation exposure at a dose of 20 Gray; ECs: Epithelial cells; KEGG: Kyoto Encyclopedia of Genes and Genomes; EMT: Epithelial-mesenchymal transition; ROS: Reactive oxygen species; Ca4-high EC: Ca4 high endothelial cells; Ca4-low EC: Ca4 low endothelial cells.

The KEGG enrichment analysis revealed that upregulated genes in IM were significantly enriched in multiple inflammatory-related pathways, including NF-kB and TNF (Figure S4G). In contrast, downregulated genes did not exhibit significant enrichment in any KEGG pathway. The upregulated genes in AM were enriched in prion-induced diseases, diabetic cardiomyopathy, phagocytosis, rheumatoid arthritis, oxidative phosphorylation, fluid shear stress-related atherosclerosis, amoebiasis, viral protein interaction with cytokines and their receptors, ECM-receptor interaction, and the IL-17 signaling pathway (Figure S4B). The downregulated genes in AM were enriched in lipid metabolism and atherosclerosis, protein processing in the endoplasmic reticulum, *Salmonella* infection, regulation of actin cytoskeleton, spliceosome, tight junction, platelet activation, leukocyte transendothelial migration, adherens junction, and bacterial invasion of epithelial cells (Figure S4C). The analysis of cell–cell interaction showed that Fn1-Cd44 received by AM (Figure S4D) and App-Cd74 sent by AM exhibited the strongest communication among various cell types in the upregulated signaling (Figure S4E). Similar results were found in the App-Cd74 received by IM (Figure S4H) and Cxcl1-Cxcr2 and Cxcl2-Cxcr2 sent by IM (Figure S4I).

### Specific subtypes of monocytes exhibit higher inflammation and potential cytokine release after radiation

Monocytes are pivotal in lung injury, participating in both inflammation and repair processes [39]. Analysis of the whole samples identified two monocyte subpopulations (Figure 1B and 1C): cMono expressed Fcnb and Cd14 (Figure 1C), and ncMono expressed Fcgr3a and Adgre4 (Figure 1C). Further analysis showed that both cMono and ncMono could be subdivided into two different subsets (Figure 5A and 5G and Figure S5A and S5K). Both the proportion of cMono and ncMono subpopulations varies greatly. For instance, cMono_C2 and ncMono_C1 were obviously decreased after radiation, while cMono_C1 and ncMono_C2 were opposite (Figure 5B and 5H). Moreover, cMono_C1 showed significantly higher inflammatory and cytokine scores after IR and cMono_C2 showed no significant change (Figure 5C), while ncMono_C1 showed significantly lower inflammatory scores and ncMono_C2 showed no significant change (Figure 5F). This implies that cMono_C1 may display enhanced responsiveness to inflammatory stimuli post-radiation, fostering an excessive immune response marked by the substantial release of cytokines, thereby fueling the inflammatory process.

The analysis of DEGs showed that *RGD1563400* (official symbol known as *Pilrb2l2*), a gene associated with inflammation, was the top one upregulated gene in cMono after radiation (Figure S5B). The KEGG analysis showed that the upregulated genes in cMono were enriched in Tuberculosis, lipid and atherosclerosis, Epstein-Barr virus infection, TNF signaling pathway, NF-kB signaling pathway, Toxoplasmosis, IL-17 signaling pathway, Th17 cell differentiation, Legionellosis, and Leishmaniasis (Figure 5D). Conversely, the downregulated genes in cMono were enriched in pathways related to amyotrophic lateral sclerosis, neurodegeneration, Parkinson’s disease, Prion disease, Huntington’s disease, ribosomes, diabetic cardiomyopathy, actin cytoskeleton regulation, chemical carcinogenesis, and oxidative phosphorylation (Figure 5E). These findings suggest a potential involvement of cMono in modulating immune and inflammatory responses as well as neurodegenerative pathways. The GO analysis showed that the upregulated genes in cMono were enriched in the cellular response to biotic stimulus, cytokine-mediated signaling pathway, and regulation of apoptotic signaling pathway, highlighting their potential involvement in immune system modulation during radiation-induced pneumonia (Figure S5C). The downregulated genes in cMono were enriched in the regulation of actin cytoskeleton organization, leukocyte cell–cell adhesion, and positive regulation of T-cell proliferation, suggesting an impact on cellular structural dynamics and immune cell activation (Figure S5D). These results suggest a comprehensive influence of cMono on immune and cellular processes in the context of radiation-induced pneumonia. Finally, to study the changes in cell interactions of monocytes after radiation, we compared the significant ligand–receptor pairs between the control and IR20Gy groups based on the upregulated genes (Figure S5E and S5F). Ligands of various collagen family members (e.g., *Col4a1, Col4a2, Col4a5, Col6a3, Col6a5, Col6a6, Col1a1,* and *Col1a2*) and receptors *Cd44*, *Scd4* were found to be active in cMono, as well as *Fn1-Cd44*.

### Radiation-triggered changes in AT2 cells hint at transdifferentiation

Epithelial cells, particularly AT2 cells, are crucial for orchestrating responses to acute and chronic lung injury [40, 41]. Following radiation exposure, the proportions of both AT1 and AT2 in lung cells increased compared to the control group (Figure 1F), while in epithelial cells the proportion of AT2 decreased and AT1 increased (Figure S5I). AT2 cells are essential for lung homeostasis due to their stem cell properties and capacity to differentiate into AT1 cells, which are pivotal for efficient air exchange [9, 42, 43]. To study the change in AT2, we used the AddModuleScore function to estimate the EMT and transdifferentiation scores in AT2. Higher EMT and transdifferentiation scores indicate that radiation may activate AT2 cells to differentiate into other cells (Figure 5I). After radiation, the upregulated genes in AT2 included *Abcb1b.1*, *Cxcl10*, *Gdf15*, and *Cxcl1*, while the downregulated genes in AT2 included *H1f4* and *Mt-atp8* (Figure S5J). Further analysis using KEGG enrichment revealed that these DEGs in AT2 cells were associated with various diseases (Figure S5G and S5H).

### Subtypes of endothelial cells exhibit highly inflammatory characteristics and production potential of ROS

After exposure to radiation, endothelial cells display DNA damage, cellular hypertrophy, increased expression of inflammatory cytokines, and impairment of barrier function [44]. We found endothelial cells have two subpopulations based on the expression of carbonic anhydrase 4 (Ca4, also known as Car4): Ca4-high ECs and Ca4-low ECs (Figures 1B and 1C and 6A and 6C). The proportion of Ca4-low ECs in lung cells increased after radiation, while Ca4-high ECs decreased (Figure 1F). However, we observed an elevation in the proportion of Ca4-low ECs and a decline in Ca4-high ECs among the endothelial cell population (Figure 6B). Ca4-low ECs exhibited higher inflammatory scores after radiation, while Ca4-high ECs had no significant change (Figure 6F). Huang et al. have found a correlation between Ca4-low ECs and neutrophil recruitment and inflammation [27]. The expression profile of genes related to neutrophil recruitment and inflammation in the two subtypes is illustrated in Figure 6D, and these genes exhibit elevated expression levels in Ca4-low ECs. Despite having a lower cytokine score, the elevated inflammatory score and robust expression of inflammatory-related genes in Ca4-low ECs indicate their notable involvement in the inflammatory response (Figures 1G and 6F). To investigate alterations in endothelial cells, we computed an EndoMT single-cell score using genes from the EMT signature in the MSigDB and noted a gradual rise in the score in the IR20Gy group, particularly evident in Ca4-low ECs (Figure 6F). These results were similar to the previous findings of mouse RILI, where endothelial cells were observed to undergo a transition toward other cells [18]. The higher scores also were found in apoptosis and ROS scores, indicating that endothelial cells have a significant relationship with apoptosis and ROS after IR (Figure 6G).

**Figure 7.**
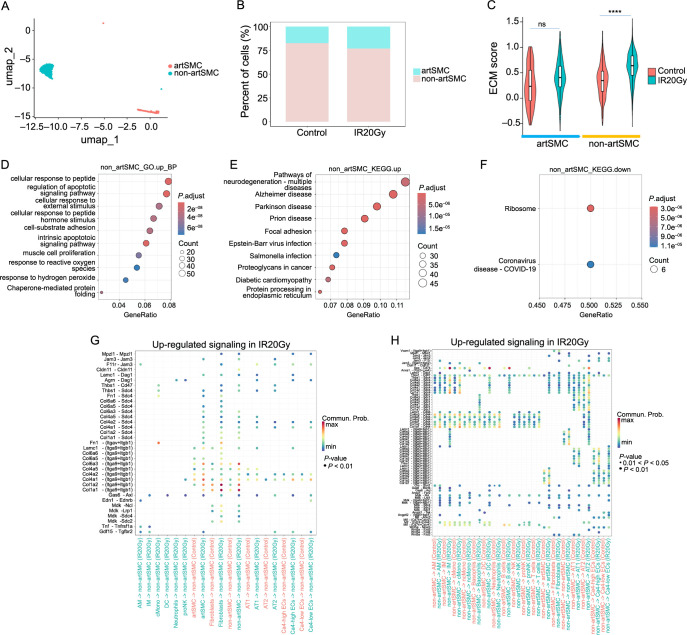
**Cellular and molecular characterization of smooth muscle cells.** (A) UMAP visualization of the two subpopulations of smooth muscle cells (artSMC and non-artSMC); (B) Changes in cell proportions of artSMC and non-artSMC in SMC; (C) The ECM score of the two cell types in each group; (D) GO terms enriched by non-artSMC upregulated genes in the IR20Gy group compared to the control group; (E and F) KEGG enriched by non-artSMC DEGs in the IR20Gy group compared to the control group; (E) Upregulated; (F) Downregulated; (G) Upregulated signaling based on upregulated genes in the IR20Gy group received by non-artSMC; (H) Upregulated signaling based on upregulated genes in the IR20Gy group sent by non-artSMC. ns, *P >*0.05; ****P <0.0001. SMC: Smooth muscle cells; ECM: Extracellular Matrix; UMAP: Uniform manifold approximation and projection; KEGG: Kyoto Encyclopedia of Genes and Genomes; IR20Gy: Ionizing radiation exposure at a dose of 20 Gray; GO: Gene Ontology; DEGs: Differentially expressed genes; artSMC: Arterial smooth muscle cells; non-artSMC: Non-arterial smooth muscle cells.

The KEGG analysis revealed that the upregulated genes in Ca4-low ECs were significantly enriched in viral infections, bacterial infections, and cellular processes such as apoptosis (Figure 6E). Conversely, the downregulated genes in Ca4-low ECs did not exhibit enrichment in any significant KEGG pathway. The enriched GO terms associated with the upregulated genes included apoptotic regulation and biological processes related to proteolysis, such as proteasome-mediated proteasomal protein catabolic process, ubiquitin-dependent protein catabolic process, and positive regulation of proteolysis (Figure S6A). This suggests that ubiquitin-mediated protein degradation is activated in endothelial cells following radiation exposure. In contrast, downregulated genes exhibited enrichment in various GO terms, including positive regulation of cell adhesion, regulation of angiogenesis, and regulation of vasculature development (Figure S6B). Furthermore, Figure S6C and S6D displayed the genes associated with the top five significant GO terms. Figure 6H and 6I and Figure S6E and S6F presented the upregulated signaling ligand–receptor pairs based on the upregulated genes in Ca4-low ECs and Ca4-high ECs, respectively. Ligand–receptor pairs App-Cd44 and Cdh5-Cdh5 were found to be active in Ca4-low ECs, while App-Cd44, Vegfa-Vegfr1, and Vegfa-Vegfr2 were found to be active in Ca4-high ECs.

### Subtypes of artSMCs show high ECM deposition characteristics

Mesenchymal cells are likely to undergo ECM deposition, promoting fibrosis [1–3]. We revealed two subtypes of SMC: artSMC-expressed Acta2 and Cspg4 and non-artSMC-expressed Acta2 not Cspg4 (Figures 1B and 1C and 7A, Figure S7F). The proportion of artSMC increased after radiation, while non-artSMC decreased (Figure 7B) in SMC. To investigate the response of SMC to fibrosis, we conducted ECM scoring (Figure 7C). There was a higher score in non-artSMC in the IR20Gy group compared to the control group, but no significant change in artSMC. It suggests that non-artSMC may have a significant impact on ECM deposition, which in turn affects the progression of pulmonary fibrosis. We utilized a Venn diagram to illustrate the overlapping genes among the upregulated genes in SMC, artSMC, non-artSMC, and ECM genes. The analysis revealed that six ECM genes—*Eln*, *Col3a1*, *Col1a1*, *Col1a2*, *Col5a2*, and *Mmp2*—were upregulated in both non-artSMC and SMC (Figure S7E). Additionally, two other ECM genes, *Fn1* and *Emilin1*, were also upregulated in SMC (Figure S7E). Notably, only one ECM gene, *Eln*, was upregulated in artSMC (Figure S7E). No ECM genes were the downregulated genes in SMC, artSMC, and non-artSMC. Additionally, *Eln* was also one of the top five upregulated genes after radiation in artSMC (Figure S7G). Unexpectedly, there were only four downregulated genes in artSMC, while there were more upregulated genes and downregulated genes in non-artSMC (Figure S7G and S7H).

The KEGG analysis showed that the upregulated genes in artSMC were enriched in various cancer pathways, including pancreatic, leukemia, thyroid, bladder, endometrial and non-small cell lung cancer, basal cell carcinoma, melanoma, glioma, and the p53 signaling pathway (Figure S7A). The downregulated genes in artSMC were not enriched in any significant pathway. The upregulated genes in non-artSMC were enriched in neurodegenerative diseases (such as Alzheimer’s, Parkinson’s, and prion diseases), cell adhesion, viral infections (Epstein–Barr virus and *Salmonella*), cancer (Proteoglycans in cancer), diabetic cardiomyopathy, and protein processing in the endoplasmic reticulum (Figure 7E). The downregulated genes in non-artSMC were enriched in the ribosome and COVID-19 (Figure 7F). The GO terms of the upregulated gene in non-artSMC involved regulating cellular responses to peptides, external stimuli, peptide hormones, and ROS, influencing processes, such as apoptosis, cell–substrate adhesion, and muscle cell proliferation (Figure 7D). The GO terms of the upregulated gene in artSMC involved the response of ketones, glucocorticoids, phosphorylation, and negative regulation of phosphorylation, phosphorus metabolic processes, and phosphate metabolic processes (Figure S7B). The downregulated genes in artSMC and non-artSMC had no significant GO term. Figure 7G and 7H and Figure S7C and S7D showed the upregulated signaling ligand–receptor pairs based on the upregulated gene in non-artSMC and artSMC respectively. Ligands-receptor pairs App-Cd44 and collagen family members (e.g., Col4a1, Col4a2, Col4a5, Col6a3, Col6a5, Col6a6, Col1a1, and Col1a2)–(Itga9+Itgb1) were found to be active in non-artSMC (Figure 7G and 7H), while App-Cd44 and collagen family members (e.g., Col4a1, Col4a2, Col4a5, Col6a3, Col6a5, Col6a6, Col1a1, and Col1a2)–(Itga1+Itgb1) in artSMC (Figure S7C and S7D).

## Discussion

In our study, we constructed a single-cell atlas of rat lungs to depict their responses to radiation. Through cell-type annotations, we identified 18 major lung cell populations using scRNA-seq technology. Our investigation unveiled ongoing transcriptional changes in each lung population following radiation exposure, sustaining the acute inflammatory phase even after only a 2-week period with a 20 Gy radiation dose. The analysis of molecular changes induced by a 20 Gy radiation dose provided insights into the pathophysiological aspects of radiation injury in the lung. The decline in immune cell proportions and the increase in stromal cell numbers post-radiation may be attributed to the higher radiosensitivity of immune cells compared to stromal cells [17]. However, when subjected to inflammatory and cytokine scoring, they exhibited higher scores post-radiation, suggesting their significant involvement in radiation pneumonia. This is particularly evident for neutrophils, monocytes, AM, and IM. These cell types may release a substantial number of cytokines, thereby either promoting or suppressing inflammation. Cao et al. [45] identified *Ebi3* as one of the top upregulated genes in LPS-induced ALI. Similarly, in our study, we also observed upregulation of *Ebi3*, which also overlaps with genes upregulated in inflammatory and cytokine responses. Moreover, the elevated expression levels of *Ebi3* post-radiation predominantly occur in cMono, Neutrophils, and IM. This suggests that *Ebi3* plays a pivotal role in acute lung injury, exerting its influence primarily through these three cellular subtypes.

Upon further subtyping of neutrophils, two distinct subgroups, cluster A and cluster B, were identified. Inflammatory response gene set scoring and cytokine gene set scoring revealed a significant increase in the scores for cluster A. KEGG and GO enrichment analyses of cluster A indicated enrichment in pathways related to positive regulation of TNF production, positive regulation of TNF superfamily cytokine production, and pathways associated with IL-1 production and TNF production. TNF and IL-1 are common proinflammatory factors, suggesting that cluster A may have a proinflammatory role. Ohms et al. [46] identified two phenotypes of neutrophils, N1 and N2, where N1-polarized neutrophils exhibit a pro-inflammatory phenotype characterized, among other factors, by elevated levels of ICAM-1 and high secretion of IFNγ-induced protein 10/CXCL10 and TNF. In our study, post-radiation neutrophils showed upregulation of *Icam1* and *Cxcl10* (see Figure S3I and S3K), predominantly in cluster A. Based on these findings, we speculate that cluster A may represent N1-polarized neutrophils. The increased proportion of cluster A after radiation suggests that radiation promotes neutrophil polarization toward the N1 phenotype. However, the mechanisms underlying the promotion of neutrophil polarization toward the N1 phenotype in response to radiation warrant further investigation. Simultaneously, we further subdivided AM, IM, cMono, and ncMono into subgroups. Some of these subgroups exhibited a significant increase in inflammatory and cytokine scores post-radiation. Curras-Alonso et al. [18] utilized single-cell sequencing to unveil proinflammatory characteristics in certain subpopulations of macrophages within mouse lung tissue post-radiation. Consistently, our study also observed similar findings. By assessing the inflammatory response scores of various subpopulations within AM and IM, we identified AM_C2, AM_C3, IM_C1, and IM_C3 as potential proinflammatory subtypes. However, their specific roles in radiation-induced pneumonia require further investigation. Yang et al. [9] employed single-cell sequencing to uncover a reduction in the number of AT2 cells in mouse lung tissue eight weeks after a single dose of 20Gy radiation. Similarly, Curras-Alonso et al. [18] also reported a decrease in AT2 cell numbers at 1–5 months post-radiation with doses of 10 or 17 Gy. However, in contrast to these findings, our study revealed a slight increase in AT2 cell numbers two weeks after a single 20-Gy radiation exposure. This discrepancy may stem from differences in the timing of our observations, focusing on the short-term effects of radiation. We speculate that radiation may initially induce an increase in AT2 cell numbers before leading to a subsequent decrease.

Numerous studies have reported radiation-induced damage to endothelial cells [5, 6]. Our analysis of the DEGs in irradiated endothelial cells revealed the enrichment of multiple pathways related to cell apoptosis and ROS. Endothelial cells exhibited high scores for both apoptosis and ROS after radiation. Similarly, the previous report showed that radiation increased ROS, causing DNA damage and endothelial cell disruption, leading to cell death [7]. Although our study observed an increase in the proportion of endothelial cells post-radiation in lung tissues, the proportions of two endothelial cell subtypes in ECs changed. Ca4-high ECs showed a decrease, while Ca4-low ECs increased in endothelial cells. Gillich et al. [47] classified endothelial cells into general capillary (gCap) and aerocyte capillary (aCap) based on Ca4 expression, where aCap was responsible for gas exchange. In our study, Ca4-high ECs and Ca4-low ECs were identified as corresponding to aCap and gCap, respectively. Ellis et al. [48] reported that lung endothelial cells could be divided into Ca4-high and Ca4 non-expressing or low-expressing subtypes, with a close relationship between Ca4-high ECs and AT1. Zhang et al. [49] further identified two subgroups within Ca4-low ECs, involving immune and developmental ECs. Our study also found that Ca4-low ECs participated in inflammatory reactions and might be involved in recruiting neutrophils, consistent with the report by Huang et al. [27]. In addition, stromal cells undergo ECM deposition, with a focus on fibroblasts and less attention on SMC changes in many reported studies [7, 18]. Interestingly, our study found a significant increase in ECM scores in SMCs after radiation, suggesting that SMCs play a crucial role in the development of RILF.

## Conclusion

In summary, our single-cell atlas of rats revealed that lung cells undergo dynamic changes in gene expression after exposure to radiation. Within this population, certain types of neutrophils were found to potentially contribute to inflammation, as evidenced by increased cytokine activity. Additionally, distinct subgroups of AM, IM, cMono, and ncMono showed a significant increase in both inflammatory and cytokine scores following radiation exposure. Furthermore, stromal cells were shown to have a significant impact on the response to radiation in the lungs. Alterations in endothelial cells also indicated potential implications for modulating the immune response. Moreover, the role of SMCs in RILF was found to be significant. These findings broaden insights into RILI, offering avenues for further exploration and potential clinical applications.

## Supplemental data

Supplementary data can be found at the following links:

https://www.bjbms.org/ojs/index.php/bjbms/article/view/10357/3205

Table S1: https://www.bjbms.org/ojs/index.php/bjbms/article/view/10357/3203

Table S2: https://www.bjbms.org/ojs/index.php/bjbms/article/view/10357/3204

## Data Availability

Our study data can be obtained from the corresponding author upon reasonable request.
